# Influence of gender on age-associated in-hospital mortality in patients with sepsis and septic shock: a prospective nationwide multicenter cohort study

**DOI:** 10.1186/s13054-023-04515-5

**Published:** 2023-06-11

**Authors:** Ryoung-Eun Ko, Danbee Kang, Juhee Cho, Soo Jin Na, Chi Ryang Chung, Sung Yoon Lim, Yeon Joo Lee, Sunghoon Park, Dong Kyu Oh, Su Yeon Lee, Mi Hyeon Park, Haein Lee, Chae-Man Lim, Gee Young Suh, the Korean Sepsis Alliance (KSA) investigators, Kyeongman Jeon, Yeon Joo Lee, Sang-Bum Hong, Young-Jae Cho, Jeongwon Heo, Jae-myeong Lee, Kyung Chan Kim, Youjin Chang, Sang-Min Lee, Suk-Kyung Hong, Woo Hyun Cho, Sang Hyun Kwak, Heung Bum Lee, Jong-Joon Ahn, Gil Myeong Seong, Song-I. Lee, Tai Sun Park, Su Hwan Lee, Eun Young Choi, Jae Young Moon

**Affiliations:** 1 Department of Critical Care Medicine, Samsung Medical Center, Sungkyunkwan University School of Medicine, Seoul, Republic of Korea; 2 Center for Clinical Epidemiology, Samsung Medical Center, Seoul, Republic of Korea; 3 Department of Clinical Research Design and Evaluation, SAIHST, Sungkyunkwan University, Seoul, Republic of Korea; 4 Department of Pulmonary and Critical Care Medicine, Seoul National University Bundang Hospital, Seongnam, Republic of Korea; 5 Department of Pulmonary, Allergy and Critical Care Medicine, Hallym University Sacred Heart Hospital, Anyang, Republic of Korea; 6 Division of Pulmonology and Critical Care Medicine, Department of Internal Medicine, Asan Medical Center, University of Ulsan College of Medicine, Seoul, Republic of Korea; 7 Division of Pulmonary and Critical Care Medicine, Department of Medicine, Samsung Medical Center, Sungkyunkwan University, Seoul, Republic of Korea

**Keywords:** Age, Gender, Sepsis, Septic shock, Sex

## Abstract

**Background:**

Numerous epidemiological studies investigating gender-dependent clinical outcomes in sepsis have shown conflicting evidence. This study aimed to investigate the effect of gender on in-hospital mortality due to sepsis according to age group.

**Methods:**

This study used data from the Korean Sepsis Alliance, an ongoing nationwide prospective multicenter cohort from 19 participating hospitals in South Korea. All adult patients diagnosed with sepsis in the emergency departments of the participating hospitals between September 2019 and December 2021 were included in the analysis. Clinical characteristics and outcomes were compared between male and female. Eligible patients were stratified by age into 19–50 years, 50–80 years, and ≥ 80 years old individuals.

**Results:**

During the study period, 6442 patients were included in the analysis, and 3650 (56.7%) were male. The adjusted odds ratio (OR) [95% confidence interval (CI)] for in-hospital mortality for male compared with female was 1.15 (95% CI = 1.02–1.29). Interestingly, in the age 19–50 group, the risk of in-hospital mortality for males was significantly lower than that of females [0.57 (95% CI = 0.35–0.93)]. For female, the risk of death remained relatively stable until around age 80 (*P* for linearity = 0.77), while in males, there was a linear increase in the risk of in-hospital death until around age 80 (*P* for linearity < 0.01). Respiratory infection (53.8% vs. 37.4%, *p* < 0.01) was more common in male, whereas urinary tract infection (14.7% vs. 29.8%, *p* < 0.01) was more common in female. For respiratory infection, male had significantly lower in-hospital mortality than female in the age 19–50 groups (adjusted OR = 0.29, 95% CI = 0.12–0.69).

**Conclusions:**

Gender may influence age-associated sepsis outcomes. Further studies are needed to replicate our findings and fully understand the interaction of gender and age on the outcomes of patients with sepsis.

**Supplementary Information:**

The online version contains supplementary material available at 10.1186/s13054-023-04515-5.

## Background

Despite major medical advances, sepsis continues to affect a large number of patients and remains one of the leading causes of hospital death worldwide [1–4]. Over the past several decades, numerous epidemiological studies on sepsis have been published. However, the epidemiology of sepsis remains unclear as sepsis is a heterogeneous syndrome.

Sepsis is characterized by a systemic dysregulated host response to infection [5]. Therefore, most of the studies on risk factors for mortality in sepsis focus on a patient’s predisposition to infection and developing organ dysfunction. Besides age, immunosuppressive disease, and diabetes, which are well-established patient susceptibility risk factors, gender may also influence the clinical outcomes of sepsis [6]. The impact of gender on sepsis has been explored by various clinical and epidemiological studies in the past decade [7, 8]. Some epidemiological studies have found a lower incidence of sepsis in females than in males [9–11]. However, the evidence for gender-dependent clinical outcomes in sepsis is inconsistent among numerous observational clinical studies, and there is no clear data on how gender influences the outcome once sepsis occurs [7, 8].

This study aimed to investigate the effect of gender on in-hospital mortality due to sepsis using data from a nationwide prospective multicenter cohort in South Korea. To better understand the effects of gender, subgroup analyses were performed to assess whether the effects of gender differed significantly across age groups.

## Materials and methods

### Study design and population

This study used data from a prospective cohort of the Korean Sepsis Alliance, an ongoing nationwide prospective multicenter study evaluating the clinical characteristics, management, and outcomes of sepsis and septic shock patients. Patients were enrolled from 19 participating hospitals between September 2019 and December 2021. The detailed protocol for patient enrolment and data collection have been previously described [12]. Patients were included if they were 19 years of age or older and were diagnosed with sepsis or septic shock in the emergency department. The diagnoses of sepsis and septic shock were based on the third International Consensus Definitions for Sepsis and Septic Shock (Sepsis-3) [5]. Patients were excluded if sepsis was first detected in the general ward.

The study was approved by the institutional review boards of each participating hospital, including Samsung Medical Center (IRB No. 2018-05-108). The requirement for informed consent was waived owing to the observational nature of the study.

### Data collection

Data on demographic characteristics, coexisting conditions, severity of illness, and treatment were collected by trained nurses [12]. In this study, we used demographic factors, including age, gender, body mass index (BMI), and clinical characteristics, including Charlson comorbidity index, illness severity using the initial Sequential Organ Failure Assessment (SOFA) score at time zero [13], recognition of sepsis by physicians in the emergency department, site of infection (e.g., respiratory, abdominal, urinary or skin/soft tissue), admission source (community, nursing home, or hospital actuated), identification of the pathogen, microbiological type (Gram-positive bacteria, Gram-negative bacteria, virus, fungus, and tuberculosis), appropriateness of antibiotics, Surviving Sepsis Campaign (SCC) bundle and its individual component completion at 3 h [14], surgical or radiologic intervention for source control, admission/transfer to intensive care unit (ICU), and length of hospital stay. The appropriateness of antibiotics was determined according to the drug susceptibility test results or the guideline recommendations [15].

The primary endpoint was in-hospital mortality rate. The main exposure was gender. Since age was also a strong confounding factor, we stratified the patients by age.

### Statistical analysis

Baseline characteristics of patients were summarized as numbers and proportions for categorical variables and mean with standard deviation or median with interquartile range (IQR, 25–75th percentiles) for continuous variables. Age was stratified into 19–50, 50–80, and > 80 years based on the average age of menopause and the influence of aging-related frailty [16, 17].

We calculated the odds ratio (OR) with 95% confidence intervals (CI) for in-hospital mortality by gender using a conditional logistic regression model, with the hospital as a matching variable to account for the potential confounding effect of the hospital. To control for other potential confounding factors, a literature review was conducted to identify relevant variables. Age and Charlson scores were selected to represent patients’ baseline condition. The year of study was included to control the time effect, including the effect of the Coronavirus disease 2019 pandemic and technical or treatment changes with time. Initial SOFA score was selected to adjust for the baseline severity of the disease. To address multicollinearity issues, we examined the correlation between variables and selected the most comprehensive variables from those with high correlations. The final model was adjusted for age, Charlson comorbidity score, initial SOFA score, presence of septic shock, site of infection, type of infection, ICU admission/transfer, and year of infection. We also performed a subgroup analysis to confirm that the association between gender and in-hospital mortality was consistent across the three age groups. For this analysis, we did not adjust for age. Patients were further divided according to presence and absence of septic shock. Another subgroup analysis to evaluate whether the association between gender and mortality was consistent across different sites of infection was also performed.

To further investigate the influence of age by gender, we modelled age as a continuous variable using restricted cubic splines. Four knots were selected based on the model comparison by Akaike Information Criterion. To determine the optimal location of knots, Harrell's suggested knot locations recommend using the 5th, 35th, 65th, and 95th percentiles of the continuous variable [18]. Thus, we performed cubic splines for age with knots at the 5th, 35th, 65th, and 95th percentiles of our sample distributions (male: 47.5, 68, 78, and 88, respectively, and female: 47, 71, 81, and 91, respectively). We then calculated the linearity and nonlinearity of the association between age and in-hospital mortality by testing that the coefficients associated with the nonlinear components are equal to zero [19].

All tests were two-sided, and a *P* value ≤ 0.05 was considered to indicate statistical significance. All analyses were performed using SAS^®^ Visual Analytics (SAS Institute Inc., USA) and STATA version 16 (StataCorp LP, College Station, TX, USA).

## Results

**Fig. 1:**
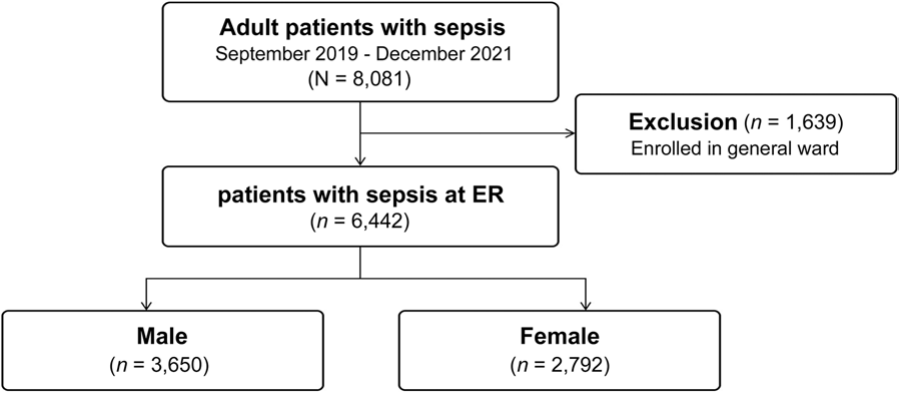
Flow diagram of the eligible study cohort from the Korea Sepsis Alliance

During the study period, 8081 patients were enrolled in the registry (Fig. 1). In this study, patients diagnosed with sepsis or septic shock in the emergency department were included, while those diagnosed in the general ward (*n* = 1639) were excluded. Finally, 6442 patients were included in the analyses (3650 male and 2792 female).

### Baseline characteristics of study patients

Of the 6442 eligible patients, 3650 (56.7%) were male, and the median age was 75 years. The baseline characteristics of the study cohort are shown in Table 1. Compared with females, males were more likely to be younger (73 [IQR, 64–81] years vs. 77 [IQR, 66–84] years, *p* < 0.01), have lower BMI (21.5 ± 4.0 vs. 22.1 ± 4.5, *p* < 0.01), and have a higher initial SOFA score (6 [IQR, 4–8] vs. 5 [IQR, 4–8], *p* < 0.01). No significant difference in the proportion of septic shock was observed between the two groups. Regarding the infection site, respiratory infection (53.8% vs. 37.4%, *p* < 0.01) was more common in males, whereas urinary tract infection (14.7% vs. 29.8%, *p* < 0.01) was more common in females. Microbiological pathogens were identified in 3822 (59.3%) patients. Among them, Gram-positive bacteria (30.7% vs. 25.1%, *p* < 0.01) and viruses (3.5% vs. 2.3%, *p* = 0.03) were more common in males, while gram-negative bacteria (72.3% vs. 77.7%, *p* < 0.01) was more common in females. The appropriateness values of initial empiric antibiotics in males and females were 88.7% and 88.3%, respectively (*p* = 0.88). The time to antibiotics administration (2 [IQR, 1–4] hours vs. 2 [IQR, 1–3] hours, *p* = 0.53) was similar for both groups. Four-hundred and ten patients (11.2%) received surgical or radiological intervention for source control in males versus 343 (12.3%) for females (*p* = 0.08). Time to source control was similar for the two groups (13 [IQR, 6–26] hours vs. 12 [IQR, 6–25] hours, *p* = 0.40). There was no significant difference between the two groups in the SCC bundle completion rate at 3 h (61.3% vs. 59.2%, *p* = 0.08). However, among individual components of the bundle, there was significant difference in lactate measurement (94.8% vs. 93.4%, *p* = 0.02). In addition, the rates of ICU admission (44.4% vs. 43.8%, *p* = 0.66) and length of hospital stay (12 [IQR, 6–21] days vs. 11 [IQR, 6–19] days, *p* = 0.30) were similar in both groups.

**Table 1:** Baseline characteristics of patients, infection, and treatments according to gender

	Male	Female	*P* value
	(*N* = 3650)	(*N* = 2792)	
Age, years	73 (64–81)	77 (66–84)	< 0.01
Body mass index, kg/m^2^	21.5 (4.0)	22.1 (4.5)	< 0.01
Charlson comorbidity index	5 (4–7)	5 (4–7)	0.01
Initial SOFA score	6 (4–8)	5 (4–8)	< 0.01
Septic shock	636 (17.4)	461 (16.5)	0.33
Sepsis suspected from the ER	1517 (41.6)	1211 (43.4)	0.14
Site of infection			
Respiratory	1962 (53.8)	1045 (37.4)	< 0.01
Abdominal	956 (26.2)	756 (27.1)	0.43
Urinary	538 (14.7)	831 (29.8)	< 0.01
Skin/soft tissue	107 (2.9)	100 (3.6)	0.14
Catheter-related	18 (0.5)	18 (0.6)	0.42
Neurologic	31 (0.8)	18 (0.6)	0.35
Type of infection			< 0.01
Community	2527 (69.2)	1790 (64.1)	
Nursing home acquired	243 (6.7)	279 (10)	
Nursing hospital acquired	494 (13.5)	432 (15.5)	
Hospital acquired	386 (10.6)	291 (10.4)	
Pathogen identification	2093 (57.3)	1729 (61.9)	
Microbiological Type (*N* = 3822)			
Gram-positive bacteria	642 (30.7)	434 (25.1)	< 0.01
Gram-negative bacteria	1514 (72.3)	1343 (77.7)	< 0.01
Virus	73 (3.5)	39 (2.3)	0.03
Fungus	123 (5.9)	102 (5.9)	0.98
M. tuberculosis	26 (1.2)	15 (0.9)	0.26
Appropriateness of initial empirical therapy		0.88	
Appropriate	3236 (88.7)	2464 (88.3)	
Inappropriate	393 (10.8)	311 (11.1)	
Not applicable	21 (0.6)	17 (0.6)	
Time to antibiotics, hours	2 (1–4)	2 (1–3)	0.53
Source control	410 (11.2)	343 (12.3)	0.19
Time to source control, hours	13 (6–26)	12 (6–25)	0.40
SSC bundle completion at 3 h	2239 (61.3)	1652 (59.2)	0.08
Measure lactate	3461 (94.8)	2608 (93.4)	0.02
Blood culture	3234 (88.6)	2491 (89.2)	0.44
Antibiotics	2377 (65.1)	1803 (64.6)	0.65
Fluid administration	3606 (98.8)	2760 (98.9)	0.83
Apply vasopressor	3506 (96.1)	2702 (97.8)	0.13
ICU admission/transfer	1620 (44.4)	1224 (43.8)	0.66
Length of hospital stay	12 (6–21)	11 (6–19)	0.30

Data are presented as mean (SD), median (interquartile range), or number (%)

*ER* emergency room; *ICU* intensive care unit; *SSC* Surviving Sepsis Campaign; *SOFA* Sequential Organ Failure Assessment

### Comparison of in-hospital mortality according to age

Among males, the proportions of patients in the age 19–50, age 50–80, and age ≥ 80 groups were 6.2%, 64.7%, and 29.1%, while among females, the corresponding proportions were 6.6%, 52.8%, and 40.5%, respectively. There were no significant differences in the baseline characteristics of patients among the three age groups, with similar differences among male and female in the whole population except for some individual components of the SSC bundle in certain age groups (Additional file 1: Table S1). SSC bundle completion at 3 h was significantly higher in males compared to females in age > 80 group (63.2 vs. 58.6%, *p* = 0.03). Lactate measurements were more frequently performed in age 50–80 group in males compared to females (95.0% vs. 93.0%, *p* < 0.01). In the age 19–50 group, blood culture was less frequently performed in males at 3 h compared to females (85.0% vs. 92.4%, *p* = 0.02).

The crude in-hospital mortality rate of the overall population was 27.7%, and the in-hospital mortality rates for males and females were 30.0% and 24.7%, respectively (*p* < 0.01). The adjusted OR (95% CI) for in-hospital mortality for males compared with females was 1.15 (95% CI = 1.02–1.29).

In the age 50–80 and age ≥ 80 groups, the adjusted ORs (95% CI) for in-hospital mortality followed the overall trend, with males showing significantly higher risk of mortality compared with females with adjusted OR values of 1.25 (95% CI = 1.07–1.47) for the age 50–80 group, and 1.10 (95% CI = 0.90–1.33) for the age ≥ 80 group. However, in the age 19–50 group, the mortality risk for males was significantly lower than that of females, with an OR of 0.57 (95% CI = 0.35–0.93) (Table 2).

**Table 2:** Odds ratios (95% confidence interval) for in-hospital mortality associated with gender

	Number of death (%)		*P-*values	Male versus female
	Male	Female		OR (95% CI)*
*All patients*				
Overall (*N* = 6442)	1095 (30.0)	690 (24.7)	< 0.01	1.15 (1.02, 1.29)
Age 19–50 (*N* = 412)	44 (19.4)	48 (26.0)	0.11	0.57 (0.35, 0.93)
Age 50–80 (*N* = 3836)	707 (29.9)	341 (23.1)	< 0.01	1.25 (1.07, 1.47)
Age > 80 (*N* = 2194)	344 (32.4)	301 (26.6)	< 0.01	1.10 (0.90, 1.33)
*P* for interaction for age				< 0.01
*Without septic shock*				
Overall (*N* = 5345)	824 (27.3)	523 (22.4)	< 0.01	1.15 (1.01, 1.31)
Age 19–50 (*N* = 340)	18 (12.8)	29 (22.3)	0.19	0.66 (0.38, 1.16)
Age 50–80 (*N* = 3142)	389 (26.6)	191 (20.9)	< 0.01	1.34 (1.12, 1.60)
Age > 80 (*N* = 1863)	179 (28.5)	159 (24.8)	0.02	1.15 (0.93, 1.43)
*P* for interaction for age				0.04
*With septic shock*				
Overall (*N* = 1097)	271 (42.6)	167 (36.2)	0.03	1.12 (0.86, 1.45)
Age 19–50 (*N* = 72)	15 (33.3)	14 (51.9)	0.12	0.45 (0.17, 1.22)
Age 50–80 (*N* = 694)	173 (40.1)	84 (32.1)	0.04	1.32 (0.95, 1.85)
Age > 80 (*N* = 331)	83 (52.2)	69 (40.1)	0.03	1.47 (0.94, 2.31)
*P* for interaction for age				0.10

For the subgroup analysis by age group, age was not adjusted for

*CI* confidence interval; *ICU* intensive care medicine; *OR* odds ratio; *SOFA* Sequential Organ Failure Assessment

*Hospital as a stratification factor in logistic models and further adjusted for age, Charlson comorbidity score (< 9 and ≥ 9), initial SOFA score, septic shock, site of infection, type of infection, ICU admission/transfer, and year

**Fig. 2:**
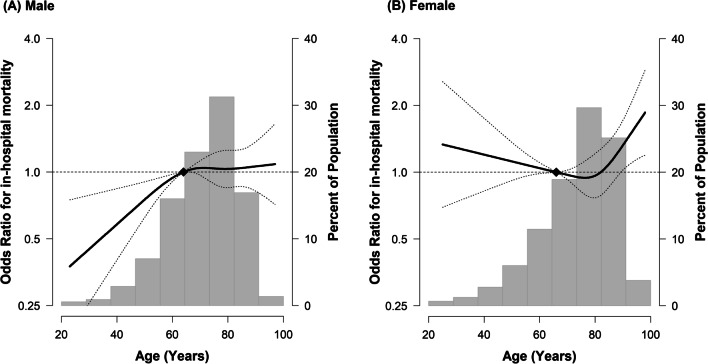
Multivariable-adjusted odds ratios (95% CI) for in-hospital death according to age in **A** male and **B** female. The curves represent adjusted odds ratios (solid line) and their 95% confidence intervals (dashed lines) for in-hospital death based on restricted cubic splines for age with knots at the 5th, 35th, 65th, and 95th percentiles of their sample distributions (male: 47.5, 68, 78, and 88, respectively, and female: 47, 71, 81, and 91, respectively). The reference value (diamond dot) is set at the 25th percentile (age 64 years for male, 66 years of female). The model is adjusted for age, Charlson comorbidity score (< 9 and ≥ 9), initial SOFA score, septic shock, site of infection, type of infection, ICU admission/transfer, and year

Restricted cubic splines used to estimate the risk of in-hospital mortality by age according to gender are shown in Fig. 2. The risk of in-hospital mortality by age showed a markedly different pattern between the two gender groups. In females, the risk of death remained relatively stable until around 80 years of age (*P* for linearity = 0.81), when the risk of death increased (*P* for linearity < 0.01). On the other hand, in male, there was a linear increase in risk of in-hospital death until around age 80 (*P* for linearity < 0.01), which seemed to plateau (*P* for linearity = 0.87).

### Comparison of in-hospital mortality according to the presence of shock

In sepsis patients without shock, in-hospital mortality rates in male and female were 27.3% and 22.4% (*p* < 0.01), respectively, with an adjusted OR of 1.15 (95% CI = 1.01–1.31). In the three age groups, males were more likely to have a higher risk of in-hospital death than females in the age 50–80 group, but the association was not significant in the age 19–50 and ≥ 80 groups.

In patients with septic shock, in-hospital mortality rates were 42.6% and 36.2% for males and females, respectively (*p* = 0.03), but adjusted OR 1.12 (95% CI = 0.86–1.45) was not statistically significant. When analyzed according to the three age groups, the adjusted OR for in-hospital death was not significantly different.

### Comparison of in-hospital mortality according to site of infection

The adjusted ORs for gender-related in-hospital mortality stratified by site of infection are shown in Table 3. In respiratory infection, males had significantly lower in-hospital mortality rates than female in the age 19–50 groups (adjusted OR = 0.29, 95% CI = 0.12–0.69). In contrast, in the age 50–80 group, males had significantly higher in-hospital mortality rates than females (adjusted OR = 1.30, 95% CI = 1.02–1.64). There was a significant interaction between age and in-hospital mortality (*p* < 0.01 for interaction). Overall, for other sites of infection, there was no significant differences in adjusted OR for in-hospital mortality between male and female patients.

**Table 3:** Odds ratios (95% Confidence interval) for in-hospital mortality associated with gender by the site of infection

Infection type	Number of death (%)		*P* values	Male versus female
	Male	Female		OR (95% CI)*
*Respiratory*				
Overall (*N* = 3007)	666 (33.9)	337 (32.3)	0.35	1.11 (0.94, 1.32)
Age 19–50 (*N* = 145)	11 (12.2)	18 (32.7)	< 0.01	0.29 (0.12, 0.69)
Age 50–80 (*N* = 3836)	413 (34.2)	147 (28.7)	0.03	1.30 (1.03, 1.64)
Age > 80 (*N* = 2194)	242 (36.5)	172 (36.0)	0.86	1.01 (0.78, 1.29)
*P* for interaction for age				< 0.01
*Abdominal*				
Overall (*N* = 1712)	260 (27.2)	178 (23.5)	0.09	1.03 (0.81, 1.31)
Age 19–50 (*N* = 132)	20 (25.0)	15 (28.9)	0.63	0.78 (0.34, 1.81)
Age 50–80 (*N* = 3142)	185 (27.1)	108 (24.4)	0.32	1.02 (0.76, 1.38)
Age > 80 (*N* = 1863)	55 (28.7)	55 (21.1)	0.06	1.07 (0.67, 1.70)
*P* for interaction for age				0.81
*Urinary*				
Overall (*N* = 1369)	101 (18.8)	131 (15.8)	0.15	1.18 (0.87, 1.60)
Age 19–50 (*N* = 72)	3 (13.0)	3 (6.5)	0.37	2.11 (0.37, 12.09)
Age 50–80 (*N* = 694)	55 (18.3)	50 (13.0)	0.05	1.47 (0.95, 2.27)
Age > 80 (*N* = 331)	43 (20.0)	78 (19.5)	0.88	0.91 (0.59, 1.41)
*P* for interaction for age				0.24
*Others*				
Overall (*N* = 292)	50 (32.1)	35 (25.7)	0.24	1.11 (0.59, 2.06)
Age 19–50 (*N* = 37)	6 (22.2)	2 (20.0)	0.88	0.86 (0.12, 6.11)
Age 50–80 (*N* = 180)	31 (31.3)	19 (23.5)	0.24	1.14 (0.53, 2.44)
Age > 80 (*N* = 331)	13 (43.3)	14 (31.1)	0.28	1.01 (0.31, 3.32)
*P* for interaction for age				0.96

For the subgroup analysis by age group, age was not adjusted for

*CI* confidence interval; *ICU* intensive care medicine; *OR* odds ratio; *SOFA* Sequential Organ Failure Assessment

*Hospital as a stratification factor in logistic models and further adjusted for age, Charlson comorbidity score, initial SOFA score, septic shock, site of infection, type of infection, ICU admission/transfer, and year

## Discussion

In this study, males had higher overall adjusted in-hospital mortality rates than females. Interestingly, the influence of gender on in-hospital mortality differed among the age groups. In the age 19–50 group, males had significantly lower adjusted odds for in-hospital mortality than females, while the reverse was true in older patients. In addition, in the restricted cubic splines, there was a linear increase in mortality in males up to age 80 years, while in females, the effect of age on in-hospital mortality seemed to be stable over the same age range. When stratified by site of infection, the interaction between age and gender was most prominent in patients with respiratory infections.

Numerous observational studies investigating the effects of gender on mortality in patients with sepsis have reported conflicting results. While most studies showed no difference in clinical outcomes between the two genders [20–23], some reported higher mortality rates in males [10, 11, 24] and others reported the opposite results [25, 26]. In this study, we found that overall, males had higher mortality rates than females (OR = 1.24, 95% CI = 1.10–1.40). This diversity observed in gender-related mortality reported in patients with sepsis may be related to the case mix of patient populations included in different studies. One of the important clinical features that differed across the sepsis cohorts was age; the average age of the previous study cohorts ranged from 60 to 76 years [10, 22].

In this study, to investigate the influence of age on gender-related mortality, two detailed analyses were performed. One was the stratification of patients into three age groups: 19–50, 50–80, and ≥ 80 years. The other was the restricted cubic splines to investigate whether the influence of age on in-hospital mortality was similar for the two genders. Interestingly, we found that male had significantly lower in-hospital mortality rate than female in the age 19–50 group, and the results were reversed in the age 50–80 and ≥ 80 groups. In addition, the shape of the restricted cubic splines for age-associated in-hospital mortality was markedly different between the two gender groups, suggesting that the effect of age is different for each gender.

Another factor that might influence the effect of gender on the clinical outcome of sepsis patients may be the site of infection. Although the site of infection is a crucial factor related to the clinical outcomes of sepsis [27–29], many previous epidemiological studies of sepsis do not include detailed information [8]. In this study, the differential effect of age on gender-specific outcomes was most prominently seen in sepsis patients with respiratory infections, showing a statistically significant effect of age group on gender-associated differences in mortality. This may explain the difference between the results of this trial and a recent post-*hoc* analysis of the ARISE trial, which reported no difference in outcomes between the two genders. In that report, only 34.6% of patients had respiratory infections, compared with 46.7% in this study. The proportions of other infection sites were also different from those in our study.

What are the possible mechanisms underlying the differential effects of gender on age-associated in-hospital mortality observed in our study? A potential factor that could explain the difference in mortality in males and females in the age group of 19–50 years is pregnancy. Pregnancy may predispose patients to sepsis and its rapid progression due to physiological and immunological adaptations [30]. But unfortunately, we did not have information on how many female patients were pregnant at sepsis presentation. Considering that 13% of maternal death are attributed to infection or sepsis, this effect warranted further investigation [31]. Another potential biological explanation may be the influence of gonadal hormones, such as testosterone on the immune system [32]. Further studies on the possible biological mechanisms for our findings are needed.

Several studies have suggested the presence of differential care according to gender in the initial management of sepsis, which may lead to different clinical outcomes [25, 33, 34]. In these studies, males were more likely to receive individual elements of the sepsis bundle much faster than females. In this study, there were no significant differences in completion rates of the SSC bundle [14] and its individual components at 3 h except for significant higher completion rates for lactate measurement in males (Table 1). But this difference in lactate measurements is unlikely to have influenced the findings of our study because the biggest difference in lactate measurements was in the age 50–80 group (Additional file 1: Table S1) while in the age 19–50 and age > 80 groups, there was no significant difference in completion rates of lactate measurement at 3 h. Also, in the age > 80 group, completion of the SSC bundle was more frequent in males which had higher OR for adjusted mortality than females and the only component of the SSC bundle that was significantly different in the age 19–50 group was blood culture which was more frequently completed in females.

This study has several limitations. First, the significance of our findings may have been influenced by the inherent biases of the observational study design. However, we used a large database of prospectively collected data of all consecutive patients diagnosed with sepsis in the emergency room from 19 hospitals in Korea to minimize selection bias. Additionally, we attempted to adjust for important confounders, but the potential for residual confounding remains. Second, we did not have detailed information on the hormones and cytokines required to better interpret our results. Third, small sample sizes in certain types of infections may limit the ability to generalize the findings of this study to those types of infections. For example, out of 6422 sepsis patients, less than 100 patients with catheter-related infection or neurologic infection were included. The subgroup analysis by site of infection may have been underpowered. Therefore, these results should be interpreted with caution and validated by further studies with larger sample sizes for these infections. Fourth, we only included Korean sepsis patients from ED, which may limit the generalization of our findings to patients from other countries and to hospital-acquired sepsis. Although Korean Sepsis Alliance cohort comprised sepsis patients diagnosed from ED and the general ward, we decided to exclude patients enrolled from the general ward in our analysis because the enrolment process is different for these two groups in our cohort, and we wanted to use a homogenous data set for our analysis. Finally, because we did not have data on long-term outcomes, we could not evaluate the impact of gender on long-term outcomes in patients with sepsis.

Despite these limitations, this study included a large number of sepsis patients and attempted to provide detailed information on the influence of gender on age-related mortality in sepsis. Another strength of our study was that we had information available on the site of infection and were able to analyze the effect of gender on age-associated outcomes by infection sites. Further studies are required to better understand the effects of gender on sepsis outcomes according to age.

## Conclusion

Gender may influence age-associated sepsis outcomes. Further studies are needed to replicate our findings and fully understand the effects of gender and age on the outcomes of patients with sepsis.

## Supplementary Information

**Additional file 1: Table S1**. Characteristics of Study Participants by Gender According to Age Group.

## Data Availability

The data supporting the findings of this study are available upon request from the corresponding author. The data are not publicly available because of privacy or ethical restrictions.

## Abbreviations

BMI: Body mass index
CI: Confidence intervals
COVID-19: Coronavirus disease 2019
ICU: Intensive care unit
IQR: Interquartile range
OR: Odds ratio
SSC: Surviving Sepsis Campaign
SOFA: Sequential Organ Failure Assessment
