# Supplementary material for: Influence of gender on age-associated in-hospital mortality in patients with sepsis and septic shock: a prospective nationwide multicenter cohort study
Source: Crit Care. 2023 Jun 11;27:229. doi: 10.1186/s13054-023-04515-5 (PMC10257805; doi:10.1186/s13054-023-04515-5)
Supplement: Supplementary file 1 — Additional file 1: Table S1. Characteristics of Study Participants by Gender According to Age Group. [file 13054_2023_4515_MOESM1_ESM.docx]

**Additional file 1**

**Influence of Gender on Age-associated In-hospital mortality in Patients with Sepsis and Septic Shock: A Prospective Nationwide Multicenter Cohort Study**

Ryoung-Eun Ko^1†^, Danbee Kang^2,3†^, Juhee Cho^2,3^, Soo Jin Na^1^, Chi Ryang Chung^1^, Sung Yoon Lim^4^, Yeon Joo Lee^4^, Sunghoon Park^5^, Dong Kyu Oh^6^, Su Yeon Lee^6^, Mi Hyeon Park^6^, Haein Lee^6^, Chae-Man Lim^6^, and Gee Young Suh^1,7*^; On behalf of the Korean Sepsis Alliance (KSA) investigators

**Table S1.** Characteristics of Study Participants by Gender According to Age Group

|  | **Age 19 – 50** | | | **Age 50** – **80** | | | **Age ≥80** | | |
| --- | --- | --- | --- | --- | --- | --- | --- | --- | --- |
|  | **Male** | **Female** | ***P* value** | **Male** | **Female** | ***P* value** | **Male** | **Female** | ***P* value** |
|  | **(*N =* 227)** | **(*N =* 185)** |  | **(*N =* 2,361)** | **(*N =* 1,475)** |  | **(*N =* 1,062)** | **(*N =* 1,132)** |  |
| **Age, years** | 43 (38–47) | 43 (36–47) | 0.41 | 69 (62–75) | 70 (63–76) | 0.02 | 84 (82–87) | 85 (82–88) | < 0.01 |
| **Body mass index, kg/m^2^** | 23.1 (6.3) | 21.7 (5.1) | 0.02 | 21.7 (3.8) | 22.6 (4.6) | < 0.01 | 20.7 (3.7) | 22.4 (4.1) | < 0.01 |
| **Charlson comorbidity index** | 2 (0–3) | 2 (0–4) | 0.06 | 5 (4–7) | 5 (4–7) | < 0.01 | 6 (5–8) | 6 (5–7) | < 0.01 |
| Diabetes | 48 (21.1) | 40 (21.6) | 0.91 | 919 (38.9) | 578 (39.2) | 0.87 | 343 (32.3) | 424 (37.5) | 0.01 |
| Liver disease | 40 (17.6) | 27 (14.6) | 0.41 | 331 (14) | 107 (7.3) | < 0.01 | 34 (3.2) | 41 (3.6) | 0.59 |
| Solid tumor | 39 (17.2) | 59 (31.9) | < 0.01 | 972 (41.2) | 540 (36.6) | < 0.01 | 284 (26.7) | 157 (13.9) | < 0.01 |
| Hematologic cancer | 13 (5.7) | 11 (5.9) | 0.93 | 79 (3.3) | 51 (3.5) | 0.85 | 21 (2) | 21 (1.9) | 0.84 |
| AIDS | 2 (0.9) | 1 (0.5) | > 0.99 | 4 (0.2) | 1 (0.1) | > 0.99 | 1 (0.1) | 1 (0.1) | > 0.99 |
| Chronic kidney disease | 25 (11) | 25 (13.5) | 0.44 | 324 (13.7) | 168 (11.4) | 0.04 | 162 (15.3) | 130 (11.5) | < 0.01 |
| Cognitive heart failure | 8 (3.5) | 1 (0.5) | 0.05 | 125 (5.3) | 109 (7.4) | 0.01 | 75 (7.1) | 123 (10.9) | < 0.01 |
| Myocardial infarction | 9 (4) | 5 (2.7) | 0.59 | 236 (10) | 85 (5.8) | < 0.01 | 125 (11.8) | 127 (11.2) | 0.69 |
| COPD | 3 (1.3) | 1 (0.5) | 0.42 | 218 (9.2) | 70 (4.7) | < 0.01 | 144 (13.6) | 45 (4) | < 0.01 |
| Peripheral vascular disease | 3 (1.3) | 2 (1.1) | > 0.99 | 61 (2.6) | 46 (3.1) | 0.33 | 25 (2.4) | 22 (1.9) | 0.51 |
| Cerebrovascular disease | 29 (12.8) | 7 (3.8) | < 0.01 | 443 (18.8) | 268 (18.2) | 0.65 | 287 (27) | 280 (24.7) | 0.22 |
| Dementia | 0 (0) | 1 (0.5) | 0.45 | 200 (8.5) | 156 (10.6) | 0.03 | 297 (28) | 461 (40.7) | < 0.01 |
| Hemiplegia | 14 (6.2) | 4 (2.2) | 0.05 | 141 (6) | 81 (5.5) | 0.54 | 67 (6.3) | 72 (6.4) | 0.96 |
| Connective tissue disease | 5 (2.2) | 6 (3.2) | 0.55 | 56 (2.4) | 58 (3.9) | < 0.01 | 26 (2.4) | 23 (2) | 0.51 |
| Peptic ulcer disease | 10 (4.4) | 3 (1.6) | 0.11 | 88 (3.7) | 37 (2.5) | 0.04 | 34 (3.2) | 23 (2) | 0.09 |
| **Initial SOFA score** | 6 (4–9) | 5 (3–8) | 0.05 | 6 (4–8) | 5 (4–8) | < 0.01 | 6 (4–8) | 6 (4–8) | < 0.01 |
| **Septic shock** | 45 (19.8) | 27 (14.6) | 0.16 | 432 (18.3) | 262 (17.8) | 0.68 | 159 (15) | 172 (15.2) | 0.88 |
| **Sepsis suspected from ER** | 87 (38.3) | 73 (39.5) | 0.81 | 965 (40.9) | 604 (40.9) | 0.96 | 465 (43.8) | 534 (47.2) | 0.11 |
| **Site of infection** |  |  |  |  |  |  |  |  |  |
| Respiratory | 90 (39.6) | 55 (29.7) | 0.04 | 1209 (51.2) | 512 (34.7) | < 0.01 | 663 (62.4) | 478 (42.2) | < 0.01 |
| Abdominal | 80 (35.2) | 52 (28.1) | 0.12 | 684 (29) | 443 (30) | 0.48 | 192 (18.1) | 261 (23.1) | < 0.01 |
| Urinary | 23 (10.1) | 46 (24.9) | < 0.01 | 300 (12.7) | 385 (26.1) | < 0.01 | 215 (20.2) | 400 (35.3) | < 0.01 |
| Skin/soft tissue | 15 (6.6) | 9 (4.9) | 0.45 | 68 (2.9) | 58 (3.9) | 0.08 | 24 (2.3) | 33 (2.9) | 0.34 |
| Catheter-related | 5 (2.2) | 1 (0.5) | 0.23 | 11 (0.5) | 10 (0.7) | 0.39 | 2 (0.2) | 7 (0.6) | 0.12 |
| Neurologic | 7 (3.1) | 0 (0) | 0.02 | 20 (0.8) | 13 (0.9) | > 0.99 | 4 (0.4) | 5 (0.4) | > 0.99 |
| **Type of infection** |  |  | 0.25 |  |  | 0.56 |  |  | < 0.01 |
| Community | 172 (75.8) | 155 (83.8) |  | 1709 (72.4) | 1044 (70.8) |  | 646 (60.8) | 591 (52.2) |  |
| Nursing home acquired | 4 (1.8) | 2 (1.1) |  | 92 (3.9) | 64 (4.3) |  | 147 (13.8) | 213 (18.8) |  |
| Nursing hospital acquired | 16 (7) | 8 (4.3) |  | 304 (12.9) | 210 (14.2) |  | 174 (16.4) | 214 (18.9) |  |
| Hospital acquired | 35 (15.4) | 20 (10.8) |  | 256 (10.8) | 157 (10.6) |  | 95 (8.9) | 114 (10.1) |  |
| **Pathogen identification** | 128 (56.4) | 107 (57.8) | 0.77 | 1325 (56.1) | 877 (59.5) | 0.04 | 640 (60.3) | 745 (65.8) | < 0.01 |
| **Microbiological Type (*N =* 3,822)** |  |  |  |  |  |  |  |  |  |
| Gram positive bacteria | 42 (32.8) | 22 (20.6) | 0.02 | 405 (30.6) | 215 (24.5) | < 0.01 | 195 (30.5) | 197 (26.4) | 0.25 |
| Gram negative bacteria | 76 (59.4) | 77 (72.0) | 0.12 | 967 (73.0) | 705 (80.4) | < 0.01 | 471 (73.6) | 561 (75.3) | 0.61 |
| Virus | 13 (10.2) | 8 (7.5) | 0.47 | 47 (3.6) | 14 (1.6) | < 0.01 | 13 (2.0) | 17 (2.3) | 0.75 |
| Fungus | 11 (8.6) | 7 (6.5) | 0.56 | 75 (5.7) | 52 (5.9) | 0.79 | 37 (5.8) | 43 (5.8) | 0.99 |
| M. tuberculosis | 4 (3.1) | 2 (1.9) | 0.69 | 14 (1.1) | 7 (0.8) | 0.54 | 8 (1.3) | 6 (0.8) | 0.41 |
| **Appropriateness of initial empirical therapy** |  |  | 0.44 |  |  | 0.95 |  |  | 0.38 |
| Appropriate | 204 (89.9) | 167 (90.3) |  | 2116 (89.6) | 1317 (89.3) |  | 916 (86.3) | 980 (86.6) |  |
| Inappropriate | 21 (9.3) | 18 (9.7) |  | 228 (9.7) | 147 (10) |  | 144 (13.6) | 146 (12.9) |  |
| Not applicable | 2 (0.9) | 0 (0) |  | 17 (0.7) | 11 (0.7) |  | 2 (0.2) | 6 (0.5) |  |
| **Time to antibiotics, hours** | 2 (1-4) | 2 (1-4) | 0.15 | 2 (1 – 4) | 2 (1-4) | 0.49 | 2 (1-3) | 2 (1-4) | 0.15 |
| **Source control** | 27 (11.9) | 16 (8.7) | 0.28 | 289 (12.2) | 202 (13.7) | 0.19 | 94 (8.9) | 125 (11.0) | 0.09 |
| **Time to source control, hours** | 14 (6 -27) | 11 (5-27) | 0.81 | 11 (6-26) | 9 (5-23) | 0.04 | 14 (5-26) | 17 (6- 28) | 0.25 |
| **SSC bundle completion at 3h** | 122 (53.7) | 104 (56.2) | 0.62 | 1,446 (61.3) | 885 (60.0) | 0.44 | 671 (63.2) | 663 (58.6) | 0.03 |
| **Measure lactate** | 211 (93.0) | 168 (90.8) | 0.43 | 2,244 (95.0) | 1,371 (93.0) | <0.01 | 1,006 (94.7) | 1,069 (94.4) | 0.76 |
| **Blood culture** | 193 (85.0) | 171 (92.4) | 0.02 | 2,085 (88.3) | 1,306 (88.5) | 0.83 | 956 (90.0) | 1,014 (89.6) | 0.73 |
| **Antibiotics** | 132 (58.2) | 119 (64.3) | 0.20 | 1,535 (65.0) | 967 (65.6) | 0.73 | 710 (66.9) | 717 (63.3) | 0.08 |
| **Fluid administration** | 221 (97.4) | 183 (98.9) | 0.25 | 2,331 (98.7) | 1,463 (99.2) | 0.19 | 1,054 (99.3) | 1,114 (98.4) | 0.07 |
| **Apply vasopressor** | 216 (95.2) | 178 (96.2) | 0.60 | 2,261 (95.8) | 1,428 (96.8) | 0.10 | 1,029 (96.9) | 1,096 (96.8) | 0.92 |
| **ICU admission/transfer** | 108 (47.6) | 87 (47) | 0.91 | 1062 (45) | 669 (45.4) | 0.82 | 450 (42.4) | 468 (41.3) | 0.63 |
| **Length of hospital stay** | 12 (7–20) | 11  (5–19.5) | 0.48 | 12 (6–21) | 12 (7–21) | 0.48 | 11 (6–19) | 11 (5–18) | 0.11 |

Data are presented as mean (SD), median (interquartile range) or number (%).

*Definition of abbreviations:* AIDS = acquired immunodeficiency syndrome; COPD = chronic obstructive pulmonary disease; ER = emergency room; ICU = Intensive care unit; SSC = Surviving Sepsis Campaign; SOFA = Sequential Organ Failure Assessment.
